# The impact of negative life events on aggressive behavior in college students: the mediating role of anger rumination and hostile attribution bias

**DOI:** 10.3389/fpsyg.2026.1772056

**Published:** 2026-03-12

**Authors:** Hao Yuan, Bing Wang

**Affiliations:** School of Physical Education and Health Engineering, Taiyuan University of Technology, Jinzhong, China

**Keywords:** aggressive behavior, anger rumination, college students, hostile attribution bias, negative life events

## Abstract

**Purpose:**

This study seeks to investigate the extent to which negative life events function as predictors of aggressive behavior among college students, while further elucidating the potential mediating mechanisms involving anger rumination and hostile attribution bias. The findings provide scientific evidence for understanding and addressing the college students’ aggressive behavior.

**Methods:**

Using a convenience sample of 651 college students, data were collected via the Adolescent Life Events Checklist (ASLEC), the Chinese version of the Anger Rumination Scale (ARS), the Wariness and Hostile Attributional Bias Scale (WSAP-Hostility), and the Aggression Behavior Questionnaire (BAQ). Mediation effects were tested using the PROCESS 4.2 macro for SPSS, with 5,000 bias-corrected Bootstrap samples.

**Results:**

Negative life events showed significant positive correlations with anger rumination, hostile attribution bias, and aggressive behavior (*r* > 0.60, *p* < 0.01). The direct predictive effect of negative life events on aggressive behavior was significant (*β* = 0.602, *p* < 0.01). The independent mediating effects of anger rumination and hostile attribution bias, as well as their chain mediating effect, were all significant, with effect sizes of 0.248, 0.121, and 0.078, respectively. None of the 95% confidence intervals (CIs) for the above effects contained zero.

**Conclusion:**

Negative life events not only demonstrate a direct association with aggressive behavior among college students but also exhibit an indirect link through the mediating effects of anger rumination and hostile attribution bias. The observed statistical mediation effects may reflect culturally patterned psychological processes, offering insight into the mechanisms underlying aggressive behavior among college students and informing the cultural adaptation of mental health support for students.

## 1 Introduction

Aggressive behavior refers to an individual’s propensity to inflict harm on others, which is frequently associated with adverse emotional and physical repercussions (Archer, 2009). Within the global context, aggressive behavior among college students has emerged as a formidable public health concern as it undermines the healthy psychosocial development of college students. Approximately one-third of children and adolescents worldwide encounter frequent bullying; such experiences may persist into young adulthood, with college students potentially redirecting similar pressures into aggressive behavior (UNESCO, 2019). Notably, the expression and fundamental mechanisms of aggression exhibit variability across cultural contexts (Sun and Liu, 2021). In Chinese higher education, aggressive behavior often manifests in relational and covert forms, reflecting cultural values of interpersonal harmony and emotional reserve. These encompass verbal psychological aggression, social ostracism, and malicious speech, whereas overt physical altercations are relatively few (Chen et al., 2025; Yin et al., 2019). Evidence indicates that approximately 60% of Chinese college students report experiencing bodily or emotional harm from such practices (Ye et al., 2022), highlighting an urgent need for targeted intervention.

Negative life events denote a broad spectrum of social and environmental stressors that elicit psychological discomfort. These events correlate with significant detrimental effects on an individual’s cognition, emotions, and actions, serving as pivotal risk factors for aggressive behavior (Chang et al., 2019; Dohrenwend et al., 1978). Such conduct may also be exacerbated by emotional and cognitive processing systems (Cheng and Han, 2021; Lei et al., 2023). College students often face stress due to negative life events. If the resulting negative emotions are not addressed, they are associated with behavioral problems, including aggressive behavior. The Frustration-Aggression Theory posits that stressors can elicit aggressive responses by inducing frustration (Berkowitz, 1989). Furthermore, stress theory suggests that negative life events not only relate to negative emotions but also correlate with significant obstructions to personal development, resulting in various adaptation challenges (Premkumar et al., 2020). The combined influence of internal and external factors, including psychological immaturity, cultural conflict, heightened competitive pressure, are linked to an increased likelihood of aggressive behavior manifesting among contemporary students (Hu, 2022; Luo, 2010; Yi et al., 2022). Thus, clarifying the connections between events and behaviors is essential for intervention.

Anger rumination is characterized as an unconscious and repetitive cognitive process, involving the persistent contemplation of the proximal causes and distal repercussions episodes that elicit anger. This process is associated with an increased tendency to interpret others’ behaviors as hostile, thereby potentially lowering the threshold for aggressive behavior (Bushman et al., 2005; Wu et al., 2024). Within the Chinese cultural framework emphasizing harmony, college students may be more likely to internalize anger through anger rumination rather than through direct expression (Ford and Mauss, 2015). Moreover, anger rumination frequently co-occurs with irritability in interpersonal conflicts (Gao et al., 2021; Ruddle et al., 2017). The “emotional internalization-cognitive immersion” pattern positions anger rumination as a cognitive mechanism for the perpetuation of emotions. Negative life events are linked to higher levels of anger, which in turn are correlated with increased rumination (Robinson and Alloy, 2003) and intensifying aggressive tendencies (Denson et al., 2011). Research indicates that negative life events are positively correlated with ruminative thinking (Yao et al., 2022). The transactional model of stress (Folkman et al., 1986) further explains that when individuals appraise events as threatening, they may engage in persistent anger-related cognition, forming anger rumination. Anger rumination, as a subtype of ruminative thinking, requires further exploration of its specific pathways and characteristics within the Chinese cultural context.

Hostile attribution bias refers to the tendency of individuals to interpret ambiguous intentions of others as malevolent (Su et al., 2021). It serves as a crucial predictor of aggressive behavior (Zhu and Xia, 2021) and is a fundamental cognitive component in the development of hostility. During automatic processing, this bias is associated with heightened monitoring for threat, which correlates with aggressive reactions (Zhang and Miao, 2019). According to the Social Information Processing Model (Torres et al., 2016), individuals with a tendency toward hostile interpretations are more likely to demonstrate aggressive behavior in uncertain circumstances. In Chinese society, which values “relationships,” “face,” and “interpersonal harmony,” individuals are particularly sensitive to others’ intentions. Negative life events can increase susceptibility to concerns regarding social reputation, leading to a heightened propensity for negative perceptions - a form of “relationship anxiety” linked to a psychological justification for violence. In this context, individuals may activate aggression-related scripts, which are associated with aggressive behavior (Gagnon et al., 2024).

Anger rumination and hostile attribution bias represent the primary cognitive mechanisms for aggressive behavior (Chen, 2018) that develop in response to negative life events. The two variables are highly interrelated and demonstrate mutual predictability. Research shows that anger rumination greatly predicts hostile attribution bias (Peled and Moretti, 2010), while also increasing hostile interpretations and weakening cognitive control (Denson et al., 2011; Wilkowski and Robinson, 2010). Evidence from cross-lagged analysis further supports a bidirectional predictive relationship between these two constructs (Zhu and Xia, 2021). Accordingly, such empirical foundations suggest that these variables can constitute a sequential mediation pattern, wherein negative life events are linked to aggressive behavior through emotional and cognitive pathways.

**Figure 1:**
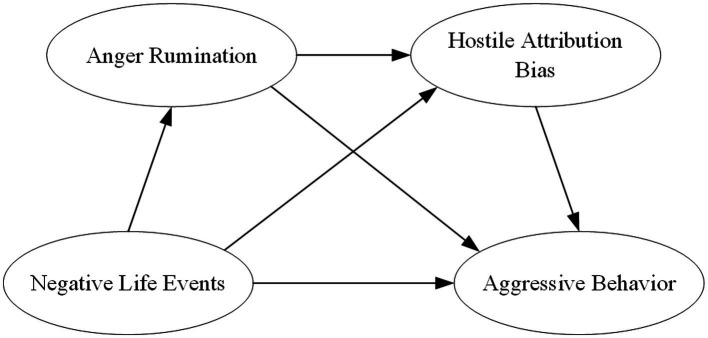
The proposed chained mediation model.

Existing research concerning aggressive behavior among college students has predominantly concentrated on single-level determinants (such as individual or family characteristics) (Hong et al., 2023) and specific behavioral manifestations. Consequently, there remains a notable lack of systematic investigation into the interactive mechanisms bridging underlying emotional states and cognitive processes. Negative life events represent a multidimensional construct encompassing diverse stressors across academic, interpersonal, familial, and economic domains. In contrast to traditional single-factor studies, the research offers a more nuanced perspective, particularly by breaking new ground in delineating the differential mechanisms that underlie various forms of aggressive behavior. Furthermore, grounded in established theoretical frameworks–including Frustration-Aggression Theory, Social Information Processing Model, and General Aggression Model, this study aims to conduct a contextualized examination of the psychological mechanisms linking negative life events to aggressive behavior within the Chinese college students. This study seeks to validate and refine the operational patterns of the “emotion-cognition” sequential mediation pathway among Chinese cultural context, specifically evaluating the chain-mediating roles of anger rumination and hostile attribution bias. Such a research orientation represents an empirical extension of existing aggression theories to non-Western educational settings, while simultaneously establishing localized foundations for future cross-cultural comparative studies and targeted intervention practices. Based on the theoretical integration described above, the following hypotheses are proposed (Figure 1):

*H1*: Negative life events demonstrate a significant positive predictive relationship with aggressive behavior among college students.

*H2*: Anger rumination serves as a mediator in the relationship between negative life events and aggressive behavior.

*H3*: Hostile Attribution Bias mediates the association between negative life events and aggressive behavior.

*H4*: Anger rumination and hostile attribution bias exert a chain-mediated role in the predictive link between negative life events and aggressive behavior.

## 2 Methods

### 2.1 Investigation process

From August 10 to September 20, 2025, the research team conducted a survey among college students in regular higher-education institutions across the country. A total of 800 students were selected through convenience sampling. The survey employed both online and offline questionnaires. Before the commencement of the study, we fully briefed all potential participants on the research purpose and procedures, and assured them of the confidentiality of their personal information. Upon obtaining their informed consent, we provided students with either paper questionnaires or online survey links for self-administration. To ensure high-quality data, we promptly distributed and collected the questionnaires. After screening out invalid responses, such as duplicate submissions and blank questionnaires, 651 valid responses were retained, including 384 online responses and 267 paper-based responses. This yielded a final validity rate of 81.3%. Convenience sampling has inherent limitations, as it tends to result in a relatively homogeneous sample, which is not conducive to comprehensive exploration. Although we diversified our sample by incorporating both online and paper questionnaires and covering various academic disciplines, potential sampling biases and limited randomness could not be eliminated. Therefore, these factors must be considered when evaluating the generalizability of our findings. In future research, more rigorous sampling methods should be adopted to verify the results obtained in this study.

### 2.2 Participants

Participants were aged 17 to 26 years (*M* = 20, *SD* = 1.44). The sample consisted of 363 males (55.8%) and 288 females (44.2%), categorized into five academic disciplines: liberal arts (*n* = 118, 18.1%), science (*n* = 161, 24.7%), engineering (*n* = 164, 25.2%), art (*n* = 79, 12.1%), and physical education (*n* = 129, 19.8%).

### 2.3 Measures

#### 2.3.1 Adolescent self-rating life events check list

Negative life events were measured using the Adolescent Life Events Scale (Liu et al., 1997). This 27-item scale assesses six dimensions, with items rated on a 5-point Likert scale (1 = never, 5 = always). The scale demonstrated high internal consistency reliability (Cronbach’s *α* = 0.957), and confirmatory factor analysis supported its construct validity.

#### 2.3.2 Anger rumination scale

Anger rumination was measured using the Anger Rumination Scale (Luo and Liu, 2017), a 19-item instrument assessing four dimensions: post-event anger, revenge thoughts, anger memory, and understanding of causes. Items were rated on a 4-point Likert scale ranging from 1 to 4. The scale demonstrated high internal consistency reliability (Cronbach’s *α* = 0.952), and confirmatory factor analysis supported its construct validity.

#### 2.3.3 Hostile attribution Bias scale

Hostile attribution bias was measured using the Word-Sentence Association Paradigm Hostility Scale (WSAP-Hostility Scale) (Dillon et al., 2016). The scale consists of 16 items, each rated on a 6-point Likert scale ranging from 1 (not at all related) to 6 (completely related). A composite score was calculated by averaging the ratings, with higher scores indicating greater hostile attribution bias. The scale demonstrated high internal consistency reliability (Cronbach’s α = 0.957), and confirmatory factor analysis supported its construct validity.

#### 2.3.4 Aggressive behavior questionnaire

The aggression measure utilized in this study was the Chinese version of the Brief Aggression Questionnaire (BAQ), originally developed by Webster et al. (2015) through the selection of 12 items with the highest factor loadings from the full Buss–Perry Aggression Questionnaire (Buss and Perry, 1992; Webster et al., 2015). The Buss–Perry Aggression Questionnaire has been widely adapted, revised, and employed in Chinese contexts, demonstrating strong reliability and validity (Li et al., 2011; Lv et al., 2013). In the present study, we administered the Brief Aggression Scale as culturally adapted and translated into Chinese (Teng et al., 2017). This 12-item scale assesses four dimensions of aggression: physical aggression, verbal aggression, anger, and hostility. Each item is rated on a 5-point Likert scale ranging from 1 to 5, with total scores computed by summing all item responses to reflect the overall level of aggressive tendencies. In this sample, the scale demonstrated good internal consistency reliability (Cronbach’s *α* = 0.811), and confirmatory factor analysis supported its construct validity.

### 2.4 Analysis

SPSS 26.0 was used for preliminary statistical analyses, and AMOS 24.0 was employed to conduct structural equation modeling. Mediation effects, including chain mediations, were tested using Model 6 of the Hayes PROCESS macro for SPSS with 5,000 bias-corrected bootstrap samples to estimate 95% confidence intervals (CIs).

## 3 Results

### 3.1 Common method Bias test

To assess the potential influence of common method variance (CMV), a Harman’s single-factor test was conducted using confirmatory factor analysis (CFA) (Podsakoff et al., 2003). A single-factor model (Model 1), in which all observed indicators were loaded onto a common latent factor, was compared against the hypothesized measurement model (Model 2) (Table 1). The chi-square difference test revealed that the hypothesized model fit the data significantly better than the single-factor model (Δ*df* = 5, Δ*χ^2^* = 1230.267, *p* < 0.001), indicating no serious common method bias in this study.

**Table 1:** Comparative analysis of confirmatory factor models for common method bias.

Model	*χ^2^*	*df*	∆*χ^2^*	∆*df*	*p*
Model 1	1452.328	77	1230.267	5	<0.001
Model 2	222.061	72			

### 3.2 Descriptive statistics

#### 3.2.1 Comparison of scores on various scales among college students of different genders

Table 2 showed that female college students scored significantly higher than male college students on hostile attribution bias (*t* = 3.449, *p* = 0.001).

**Table 2:** Comparison of scale scores between male and female college students.

Variable	Sample size	Negative life events	Anger rumination	Hostile attribution bias	Aggressive behavior
Male	363	1.3 ± 0.9	1.8 ± 0.6	2.7 ± 1.1	2.5 ± 0.7
Female	288	1.4 ± 1	1.9 ± 0.7	3 ± 1.1	2.7 ± 0.7
*t*		0.872	2.209	3.449	2.31
*p*		0.384	0.028	0.001	0.021

#### 3.2.2 Comparison of scores across dimensions of the aggression behavior scale

Among the aggression subscales, verbal aggression had the highest mean score (*M* = 2.824, *SD* = 0.805), while physical aggression had the lowest (*M* = 2.279, *SD* = 0.874), indicating that Chinese college students exhibited less direct physical aggression (Table 3).

**Table 3:** Comparison of scores across dimensions of college students’ aggressive behavior scale.

Project	Minimum value	Maximum value	Average score of items	Standard deviation	Variance	Ranking
Dimension 1: Physical Attack	1	5	2.279 ± 0.034	0.874	0.765	4
Dimension 2: Verbal Abuse	1	5	2.824 ± 0.032	0.805	0.648	1
Dimension 3: Anger	1	5	2.686 ± 0.028	0.716	0.513	2
Dimension 4: Hostility	1	12.67	2.561 ± 0.040	1.010	1.02	3
Aggressive behavior	1	4.5	2.584 ± 0.027	0.680	0.463	

### 3.3 Correlation analysis

Pearson correlation analysis (Table 4) revealed that all variables were significantly and positively correlated with each other (*r* > 0.6, *p* < 0.01).

**Table 4:** Descriptive statistics and correlation coefficients for each variable.

Variable	*M*	*SD*	1	2	3	4
1 Negative life events	1.323	0.961	1			
2 Anger rumination	1.87	0.663	0.728**	1		
3 Hostile attribution bias	2.818	1.122	0.604**	0.60**	1	
4 Aggressive behavior	2.584	0.680	0.614**	0.641**	0.626**	1

M is mean value; SD is standard deviation. *p < 0.05, **p < 0.01, ***p < 0.001. same applies below.

### 3.4 The impact of negative life events on aggressive behavior

#### 3.4.1 The direct effect of negative life events on aggressive behavior

As Table 5 shows, linear regression analysis indicated a positive predictive relationship between the two variables. Bootstrap testing revealed a direct effect value of 0.533, with a 95% confidence interval ranging from 0.467 to 0.603.

**Table 5:** Regression analysis of negative life events and aggressive behavior.

Outcome	Independent variable	*β*	*t*	*R*	*R^2^*	*F*
Aggressive behavior	Gender	−0.044	−1.364	0.626	0.392	83.17***
	Address	−0.033	−1.064			
	Major	−0.095	−3.002			
	Grade	0.013	0.399			
	Negative life events	0.602	19.438			

#### 3.4.2 The chain-mediated effect of anger rumination and hostile attribution Bias

Adding anger rumination and hostile attribution bias as mediators, chained mediation was conducted using Hayes’ SPSS PROCESS 4.2 Model 6. The results showed that Negative life events positively predicted anger rumination (*β* = 0.503, *p* < 0.01), hostile attribution bias (*β* = 0.415, *p* < 0.01), and aggressive behavior (*β* = 0.139, *p* < 0.01); anger rumination positively predicted hostile attribution bias (*β* = 0.577, *p* < 0.01) and aggressive behavior (*β* = 0.310, *p* < 0.01); and hostile attribution bias positively predicted aggressive behavior (*β* = 0.197, *p* < 0.01).

**Figure 2:**
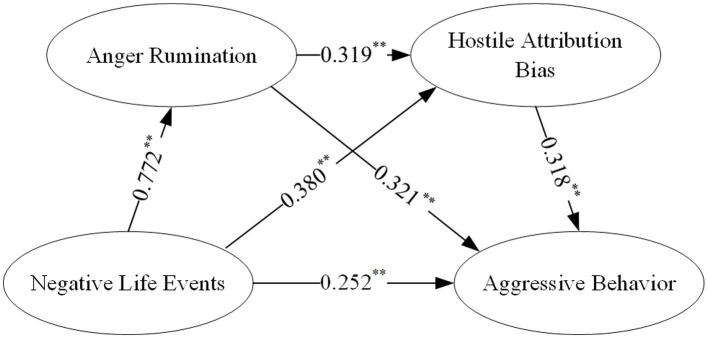
Chain mediator model figure.

By constructing a chained mediating variable structural equation model incorporating anger rumination and hostile attribution bias using AMOS 24.0 (Figure 2), the model demonstrated good fit: *χ^2^/df* = 3.34, CFI = 0.979, TLI = 0.974, IFI = 0.979, GFI = 0.948, RMSEA = 0.06.

The results of the chain mediation analysis indicate (Table 6) that negative life events exert a significant total indirect effect on college students’ aggressive behavior, with an effect size of 0.447 and a 95% confidence interval that does not include zero. The specific pathways are as follows: Path 1 (negative life events → anger rumination → hostile attribution bias → aggressive behavior) exhibited a significant mediating effect, with an effect size of 0.078 accounting for 11.2% of the total effect; Path 2 (negative life events → anger rumination → aggressive behavior) had a significant mediating effect with an effect size of 0.248, accounting for 35.5% of the total effect; Path 3 (negative life events → hostile attribution bias → aggressive behavior) demonstrated a significant mediating effect with an effect size of 0.121, accounting for 17.3% of the total effect.

**Table 6:** Results of the chain mediation effect test for anger rumination and hostile attribution bias.

Path relationship	Point estimate	Effect proportion	*SE*	95% CI
Path 1	0.078	11.20%	0.012	[0.046, 0.121]
Path 2	0.248	35.50%	0.028	[0.143, 0.335]
Path 3	0.121	17.30%	0.042	[0.077, 0.172]
Total indirect effect	0.447	64%	0.055	[0.345, 0.546]

Path 1 (negative life events → Anger Rumination → hostile attribution bias → aggressive behavior); Path 2 (negative life events → Anger Rumination → aggressive behavior); Path 3 (negative life events → hostile attribution bias → aggressive behavior).

### 3.5 Dimension-specific mediation analysis of aggressive behavior

Table 7 presented the results of the dimensional tests of the chain mediation model. Negative life events positively predicted all dimensions of aggressive behavior. After accounting for the mediators, the direct effects remained statistically significant but were substantially reduced, whereas the total indirect effects were considerable across all four dimensions. Specifically, the combined indirect effects accounted for 65.6% and 79.4% of the variance in verbal aggression and hostility, respectively. In contrast, physical aggression showed a direct effect of 44.1%. Path analysis further revealed that the chain mediation pathways were significant across all dimensions, with the largest effect size observed for hostility (*β* = 0.074) and the smallest for anger (*β* = 0.037).

**Table 7:** Chain mediation effect analysis of negative life events on different dimensions of aggressive behavior.

Aggressive behavior dimension	Total effect	Direct effect	Total indirect effect	Mediating pathway of anger rumination	Mediating pathway of hostile attribution bias	Chain mediation path
Physical aggression	0.522	0.230	0.291	0.154	0.08	0.056
	[0.464, 0.579]	[0.148, 0.313]	[0.217, 0.366]	[0.077, 0.230]	[0.047, 0.117]	[0.032, 0.080]
Verbal aggression	0.323	0.111	0.212	0.078	0.079	0.055
	[0.264, 0.383]	[0.023 0.199]	[0.136, 0.283]	[0.010, 0.140]	[0.048, 0.112]	[0.031, 0.085]
Anger	0.356	0.123	0.233	0.142	0.053	0.037
	[0.306, 0.407]	[0.049, 0.196]	[0.173, 0.298]	[0.091, 0.200]	[0.028, 0.058]	[0.020, 0.058]
Hostility	0.529	0.109	0.420	0.238	0.106	0.074
	[0.459, 0.599]	[0.011, 0.206]	[0.326, 0.503]	[0.172, 0.306]	[0.064, 0.150]	[0.046, 0.108]

## 4 Discussion

Research findings indicate that negative life events can predict aggressive behavior in college students, confirming Hypothesis 1 and aligning with previous studies (Yang et al., 2023). Negative life events can predispose individuals to harmful attitudes and mental distress, prompting them to resort to aggressive behaviors as a coping mechanism to manage negative emotions or alleviate stress. Those lacking adequate psychological coping skills are more prone to emotional dysregulation and tend to adopt maladaptive coping mechanisms. It is meaningful that the Chinese university students in this study predominantly exhibited subtle, relational, and covert aggression rather than overt physical aggression. This behavioral pattern can be attributed to Chinese cultural values, such as harmony and collective identity. The cumulative impact of negative life events (Burks and Martin, 1985) significantly increases the probability of psychological symptoms and externalizing behavioral problems with prolonged exposure to several occurrences. College students often face a series of negative events, such as academic stress, family conflicts, and financial problems, which can collectively exceed their psychological coping capacity. According to the frustration-aggression theory, negative life events serve as sources of dissatisfaction that incite aggressive behavior (Kruglanski et al., 2023). The Ecological Systems Theory posits that aggression results from the interplay of individual issues, family atmosphere, school climate, and societal factors (Bronfenbrenner, 1977). Thus, negative life events directly promote aggressive behavior among college students.

Hypothesis 2 has also been supported. Studies demonstrate that negative life events are significant predictors of anger rumination and its relationship with aggressive behavior in college students. This “emotional internalization” is particularly pronounced in the Chinese cultural context and can be viewed as a unique emotion regulation strategy. Anger rumination, as a subtype of rumination, serves as an intermediary mechanism explaining the association between negative life events and aggressive behavior. According to the stress response model, negative life events induce cognitive bias, reinforce negative self-schemas, and elicit cognitive distortions, ultimately leading to an automatic negative thought process (Zeng et al., 2025). Angry ruminators are more likely to dwell on their feelings rather than solve problems, especially in interpersonal contexts where anger and aggressive feelings are constantly amplified-a tendency more pronounced in individuals with high trait anger. Anger rumination is associated with prolonged and heightened anger, difficulties in emotional regulation and a greater propensity for aggressive behavior. Anger rumination is a process of emotional ferment, which involves the memory of negative events and the re-experience of anger (Bushman et al., 2005). Although attempts to stop rumination may be made, this process consumes large proportions of the cognitive resources, making it ineffective in blocking aggressive behavior (White and Turner, 2014). The associative network concept suggests a strong connection between negative events and anger, enabling even minor triggers to evoke profound anger responses. This heightened and reinforced anger is associated with increased aggressive motivation and improved rationalization, potentially serving as an emotional release mechanism. In summary, anger rumination plays a crucial role in the emotional amplification and cognitive impairment associated with the link between stress and aggressive behavior.

The findings support Hypothesis 3, which suggests that negative life events, combined with hostile attribution bias, can predict aggressive behavior. Individuals with hostile attribution bias perceive ambiguous situations as hostile and respond aggressively. The General Aggression Model (Allen et al., 2018) posits that negatively connoted negative life events generate strong negative emotions, which in turn trigger aggressive explanations of unclear inputs. The Integrated Cognitive Model (Wilkowski and Robinson, 2010) argues that negative life events lead to undesirable self-evaluations, prompting individuals to perceive others’ ambiguous actions as aggressive to preserve self-esteem. Studies indicate that hostile attribution bias is linked to specific attributes (Su et al., 2021). Individuals with high hostile attribution bias scores are likely to attribute others’ motives as malicious, justify their anger and revenge, and view aggressive behavior as the primary means of handling perceived threats, thus using aggression to address perceived injustices. Individuals with high hostility levels are more prone to engage in aggressive behavior. This study reveals that female college students are more likely to exhibit hostile attribution bias than their male counterparts. Female students may experience greater pressure to maintain harmonious interpersonal relationships during their upbringing, leading to a heightened perception of potential relational risks (Chahal et al., 2021). Perceiving ambiguous behaviors as aggression is also a psychological process that aids in early danger detection and self-protection. Women tend to be more indirectly aggressive, engaging in behaviors such as exclusion, spreading rumors, and social isolation, whereas men are more likely to resort to physical aggression (Godleski and Murray-Close, 2023). Consequently, these small social dangers may be perceived by women during the course of their development. Collectively, these findings indicated that the hostile attribution bias serves as a key cognitive mechanism linking negative life events and aggressive behavior.

The study not only validated the independent mediating roles of anger rumination and hostile attribution bias but also, more importantly, revealed a sequential mediating pathway from anger rumination to hostile attribution bias, thereby confirming Hypothesis 4. This finding supports a sequential mediational model in which external stressors trigger internal cognitive processes that subsequently predict behavioral outcomes. Specifically, negative life events among college students can precipitate cognitive responses to stress, such as anger rumination. This cognitive style is associated with the depletion of executive resources (Wang and Shi, 2024), leading to difficulties in concentration, impaired emotional regulation, and persistent angry thoughts. Such cognitive impairments, in turn, foster a biased interpretation of others’ intentions-namely, hostile attribution bias-whereby individuals struggle to make neutral judgments about ambiguous behaviors, further perpetuating this bias. As hostile attribution bias intensifies, individuals become increasingly prone to aggressive behavior. This pattern aligns with the Social Information Processing (SIP) model, which posits that cognitive factors in aggression are interconnected through scripts of hostility and anger (Torres et al., 2016). Within this framework, anger rumination acts as an amplifying factor, increasing the accessibility and intensity of hostile schemas, thereby contributing to the development and escalation of aggressive behavior. In summary, the progression from anger rumination to hostile attribution bias represents a predictive emotion-cognition-behavior cascade that elucidates how external stressors are internally transformed into aggression.

To further elucidate the relationship between negative life events and different types of aggressive behavior, this study conducted a chain mediation analysis across four distinct dimensions of aggression. The results indicate that negative life events significantly and positively predict all dimensions, although their pathways and magnitudes of influence vary. Specifically, hostility demonstrated the most pronounced indirect effect, anger exhibited relatively weaker effects, and verbal aggression, despite its lower total effect, was highly dependent on internal emotional and cognitive processes. Mediation analysis further revealed that indirect effects predominated in the verbal aggression and hostility dimensions, suggesting that these behaviors are more strongly driven by internal psychological mechanisms, such as anger rumination and hostile attribution bias, and thus exhibit distinct implicit characteristics. In contrast, while physical aggression was also influenced by mediating pathways, its direct effect remained significant. This form of aggression appears to rely more on immediate situational stimuli or rapid responses shaped by cultural norms. This finding aligns with the behavioral patterns observed among college students in Chinese cultural contexts, wherein direct physical aggression is less common, whereas verbal and relational aggression are more prevalent.

A potential concern arises regarding the conceptual overlap between the aggressiveness measurement dimension (anger and hostility) and the mediating factors (anger rumination, hostile attribution bias). Anger is an intense emotional experience that often arises when individuals feel violated, treated unfairly, or unable to fulfill their desires. Anger rumination refers to the persistent, repetitive, and passive recollection of anger-inducing events. Hostility denotes a state of mental antagonism, primarily manifested as viewing someone as an adversary and adopting a confrontational stance. Hostile attribution bias describes the tendency to temporarily interpret others’ intentions as hostile in specific situations. The role of “anger rumination” is to sustain and intensify feelings of anger, whereas “anger” on the aggressiveness scale encompasses physiological arousal and readiness for aggressive behavior, indicating an affective component. It is undeniable that anger rumination generally coincides with feelings of anger. Similarly, “hostile attribution bias” is an immediate cognitive interpretative bias associated with the initial phases of social information processing, while the “hostility” dimension signifies a more enduring, generalized cognitive-emotional disposition or personality trait. Admittedly, a fundamental cognitive theme common to both is the “negative assessment of others’ intentions”. Empirical studies significantly support this distinction. Despite potential conceptual overlap across variables, they remain distinct in their theoretical definitions, measurement content, and statistical associations.

## 5 Practical implications

Based on the previous research (Zhu et al., 2020), this study extends the research perspective from the individual or family level to encompass a broader range of negative life events. The findings not only demonstrate the respective mediating roles of anger rumination and hostile attribution bias but also reveal a serial mediation pathway between them. This leads to the construction of a chain mediation model that illustrates the progression from external stressors through internal cognitive processing to behavioral outcomes. This model deepens our understanding of the underlying mechanisms of aggressive behavior and highlights the importance of multi-level interventions.

Furthermore, it encompasses authentic stressors encountered by Chinese university students and suggests that females have a heightened hostile attribution bias. Consequently, the findings are localized and may provide recommendations for intervention. This study establishes explicit objectives for university mental health services to mitigate and manage student aggressiveness. Initially, it is crucial to identify students who have undergone substantial negative life events and to monitor their degree of rumination. Secondly, future research and practice may investigate the application of cognitive-behavioral therapy (CBT) to disrupt negative thinking sequences likely instigated by anger rumination and hostile attribution bias (Wang and Miller, 2023; Zajenkowska et al., 2019). Furthermore, mindfulness training may aid students in overcoming habitual ruminative thinking (Argungu et al., 2025), whereas attribution group counseling could facilitate non-hostile perceptions in ambiguous situations (Hudley and Graham, 1993). These intervention strategies may reduce campus hostility and promote a peaceful environment; however, their effectiveness in this setting requires additional empirical research.

By validating the “anger rumination–hostile attribution bias” chain mediation model within a Chinese cultural context, this study provides important evidence for the international academic community’s understanding of aggression mechanisms in non-Western cultures. It extends the cultural scope of current aggression theories and suggests that future cross-cultural research should investigate how cultural contexts shape the psychological pathways from stress exposure to behavioral expression. These findings enrich existing theoretical frameworks by incorporating cultural considerations, thereby providing a more nuanced scientific foundation for designing interventions that can be adapted across diverse cultural settings.

## 6 Limitations and future directions

The present study has several limitations. First, although this study focuses on the Chinese cultural context, which facilitates an in-depth examination of culturally specific mediating processes such as anger rumination, it also limits the generalizability of the findings to other cultural groups. Future research could test the proposed model across diverse cultural contexts to examine its cross-cultural applicability. Second, this study employed a cross-sectional design, which can only reveal associations among the mediating variables within the statistical model but does not allow for causal inferences or an examination of how these relationships evolve. This design also cannot rule out the possibility of reverse causality or even bidirectional interactions (e.g., aggressive behavior may, in turn, reinforce individuals’ tendencies toward anger rumination). Future research should employ longitudinal or experimental designs to more rigorously test the proposed causal pathways. Third, although the theoretical model constructed in this study is grounded in established conceptual frameworks, it does not incorporate other potentially relevant variables. The current model remains incomplete, as it does not include factors such as personality traits (e.g., neuroticism), social support, or family background. These variables may function as moderators, independent mediators, or confounding factors within the model. Future research should further integrate these variables to promote the development of a more systematic and comprehensive theoretical framework.

## 7 Conclusion

This study identifies significant associations between college students’ aggressive behavior and negative life events, anger rumination, and hostile attribution bias. Specifically, negative life events not only serve as a direct positive predictor of such behavior but also indirectly influence its occurrence through the independent mediating roles of anger rumination and hostile attribution bias, as well as through their chain mediation pathway. To systematically elucidate the formation of aggressive behavior, this study constructs an integrated theoretical model incorporating cognitive and emotional factors, aiming to deepen the understanding of its developmental pathways within a specific educational and cultural context.

Based on empirical data from Chinese college students, the model demonstrates high cultural relevance and can provide valuable insights for developing psychological health intervention strategies for this population. However, the limitations of a cross-sectional research design and the use of a single cultural sample suggest that conclusions regarding cross-cultural applicability need further validation. Additionally, the empirically tested chain mediation model identifies several key intervenable variables, suggesting that interventions targeting anger rumination and hostile attribution bias (such as cognitive-behavioral therapy, mindfulness training, and attribution retraining programs) may have potential applications in reducing aggressive behavior among college students.

## Data Availability

The raw data supporting the conclusions of this article will be made available by the authors, without undue reservation.
